# *Mycobacterium avium* subsp. *hominissuis* Infection in Horses

**DOI:** 10.3201/eid1608.100097

**Published:** 2010-08

**Authors:** Petr Kriz, Petr Jahn, Barbora Bezdekova, Mariana Blahutkova, Vojtech Mrlik, Iva Slana, Ivo Pavlik

**Affiliations:** Veterinary Research Institute, Brno, Czech Republic (P. Kriz, M. Blahutkova, V. Mrlik, I. Slana, I. Pavlik); University of Veterinary and Pharmaceutical Sciences, Brno (P. Jahn, B. Bezdekova)

**Keywords:** Bacteria, Mycobacterium avium, horses, hippotherapy, zoonoses, letter

**To the Editor:** *Mycobacterium avium* subsp. *hominissuis* infection is often detected in pigs and humans (*1**–**3*). In most cases, the main sources of this agent are environmental (*4**,**5*). During the past few years, 2 hosts infected by this agent, dogs (*6*) and pet parrots (*7*), were identified as a possible source of infection for immunocompromised humans who may have close contact with animals. We report massive *M. avium* subsp. *hominissuis* infection in 2 sibling riding-type Fjord horses from an amateur-run horse-breeding farm.

The first horse, a 2-year-old colt, was admitted to a veterinary clinic in the Czech Republic in February 2009 with diarrhea and progressive weight loss of 3 weeks’ duration. Multiple diagnostic procedures produced inconclusive results. Cyathostomosis was suspected, so moxidectin was administered twice, and prednisolone was given for 10 consecutive days. The clinical status of the horse initially improved but worsened after 3 weeks. Ultrasonographic examination of the peritoneal cavity showed a nodular mass and a nonperistaltic, thickened portion of the small intestine wall in the left ventrocranial region. Exploratory celiotomy showed enlargement of the mesenteric and colonic lymph nodes and multiple local thickenings of the small intestine wall, large colon, and cecum. The horse was euthanized. Specimens of enlarged lymph nodes and intestinal content were taken during necropsy for histopathologic and microbiologic examination. Microscopically, acid-fast rods (AFR) after Ziehl-Neelsen staining were observed, and quantitative real-time PCR (qPCR) showed 2.89 × 10^5^ and 1.47 × 10^4^ *M. avium* subsp. *hominissuis* cells per 1 g of intestinal content and mesenteric lymph node, respectively (*8*).

The second case, a 1-year-old full sister to the colt described above, was admitted in July 2009 after 1 month of lethargy, weight loss, diarrhea, and nasal discharge. Ultrasonographic examination of the abdominal cavity showed an increased amount of peritoneal fluid and nonperistaltic, corrugated, and thickened parts of the small intestine in the left caudal region. Local thickening of the jejunum and ileum were found during exploratory celiotomy; no lesions on the cecum or colon were observed macroscopically. Mesenteric lymph nodes were enlarged. Microscopically, AFR were observed, and qPCR showed 3.36 × 10^6^ *M. avium* subsp. *hominissuis* cells per 1 g of mesenteric lymph node (*8*). Treatment with clarithromycin and rifampin was begun, but the condition of the filly improved only temporarily. She was euthanized after 4 months because of progressively worsening condition. Postmortem examination showed enlarged colonic lymph nodes with small nodular lesions, hyperemia of the colon mucosa, and corrugation and thickening of the colonic wall. For further examination, samples of feces, colonic lymph nodes and wall, liver, mesenteric lymph nodes, kidney, spleen, and diaphragm were taken. Ziehl-Neelsen staining of tissue smears demonstrated AFR in different tissues. Culture examination following the described method (*2*) and qPCR confirmed *M. avium* subsp. *hominissuis* infection; quantities of this agent were 6.31 × 10^5^ and 2.47 × 10^11^ in 1 g of feces and mesenteric lymph nodes, respectively (Table).

**Table:** Detection of *Mycobacterium avium* subsp. *hominissuis* in tissues of a 1-year-old Fjord filly*

Sample source	Mycobacteria detection			qPCR	
	Microscopy	Culture		IS*1245*†	IS*901*
Feces	−	+		6.31 × 10^5^	−
Lymph node of transversal colon	+	+		1.84 × 10^9^	−
Lymph node of descending colon	+	+		5.89 × 10^9^	−
Transversal colon wall	+	+		3.98 × 10^7^	−
Descending colon wall	+	+		6.33 × 10^6^	−
Liver	−	+		NT	NT
Mesenteric lymph node	+++	+		2.47 × 10^11^	−
Kidney	−	+		NT	NT
Spleen	−	+		NT	NT
Diaphragm	−	−		8.22 × 10^4^	−

*qPCR, quantitative real-time PCR; −, negative finding; +, few acid-fast rods (AFR); NT, not tested; +++, >100 AFR (per 50 microscopic fields). †No. IS*1245* copies/g.

According to a review (*9*), infections caused by *M. avium* subsp. *hominissuis* have been described in only 6 horses until now. We presume that *M. avium* subsp. *hominissuis* infection in both these horses could have been caused by some immunodeficiency related to a genetic predisposition. The shedding of this agent in feces indicates that infected horses can also pose a health risk to humans, particularly immunocompromised persons. *M. avium* subsp. *hominissuis* infection is frequently observed in children, in whom it can cause peripheral lymphadenopathy (*10*). Currently, hippotherapy is a frequently used recreational activity in some countries for various patients, e.g., for children with cerebral palsy. Hippotherapy thus may be associated with a potential risk for humans in contact with clinically ill *M. avium* subsp. *hominissuis*–infected horses.
